# High-Resolution Ultrasound Imaging Enabled by Random Interference and Joint Image Reconstruction

**DOI:** 10.3390/s20226434

**Published:** 2020-11-11

**Authors:** Pavel Ni, Heung-No Lee

**Affiliations:** School of Electrical Engineering and Computer Science, Gwangju Institute of Science and Technology, Gwangju 61005, Korea; pni@gist.ac.kr

**Keywords:** ultrasound imaging, image resolution, wave interference, array signal processing

## Abstract

In ultrasound, wave interference is an undesirable effect that degrades the resolution of the images. We have recently shown that a wavefront of random interference can be used to reconstruct high-resolution ultrasound images. In this study, we further improve the resolution of interference-based ultrasound imaging by proposing a joint image reconstruction scheme. The proposed reconstruction scheme utilizes radio frequency (RF) signals from all elements of the sensor array in a joint optimization problem to directly reconstruct the final high-resolution image. By jointly processing array signals, we significantly improved the resolution of interference-based imaging. We compare the proposed joint reconstruction method with popular beamforming techniques and the previously proposed interference-based compound method. The simulation study suggests that, among the different reconstruction methods, the joint reconstruction method has the lowest mean-squared error (MSE), the best peak signal-to-noise ratio (PSNR), and the best signal-to-noise ratio (SNR). Similarly, the joint reconstruction method has an exceptional structural similarity index (SSIM) of 0.998. Experimental studies showed that the quality of images significantly improved when compared to other image reconstruction methods. Furthermore, we share our simulation codes as an open-source repository in support of reproducible research.

## 1. Introduction

Medical imaging refers to different tools and techniques used in hospitals for diagnostic purposes. Noninvasive imaging modalities, particularly ultrasound imaging, play an important role in the early identification of abnormalities. In this study, we are interested in improving the spatial and contrast resolution of ultrasound imaging modalities. Conventional ultrasound imaging methods use beamforming to spatially divide the region of interest (ROI) into multiple scanlines [1,2]. Each scanline is then imaged separately using a focused ultrasound pulse. Conventional ultrasound techniques that utilize beamforming are capable of achieving an image resolution that is approximately equivalent to the width of the transmitted ultrasound beam [3]. The smallest beam width is achieved at a focal point. For a central frequency of 3 MHz, the best spatial resolution is approximately equal to 1 mm [3,4]. However, owing to wave diffraction, it is impossible to achieve an infinitely narrow ultrasound beam that travels precisely along a single scanline [4,5]. As a result of the diffraction effect, the focused ultrasound pulse is reflected not only from the tissue within the desired scanline but also from the adjunct areas. The echo signals reflected from adjunct areas degrade the image resolution by corrupting it with speckle noise [6,7,8].

In the past, different techniques have been used to enhance the spatial and contrast resolution of ultrasound systems [9,10]. For instance, in [11,12], speckle reduction techniques using statistical models have been proposed. Speckle reduction techniques use mathematically modeled filters to suppress speckle noise. To achieve significant improvements in resolution, we believe that it is insufficient to modify only the signal processing (software) part without modifications to the imaging protocol (hardware). An excellent example of symbiosis between software and hardware is the introduction of contrast agents, where two parts complement each other to produce significant improvements in resolution [13,14,15]. Contrast microbubbles are used to create a spatially localized sound source that significantly improves the ultrasound resolution.

In ultrasound, coded excitation signals are a well-known technique used to improve the quality of images [16,17]. However, to the best of our knowledge, using excitation signals to generate an ultrasound field with a spatially varying property such as one presented in this paper is a relatively new research direction. In the past few years, there is a growing interest in the development of imaging systems that use unfocused ultrasound fields with spatially variant properties. In [18], an object much smaller than the wavelength of the signal is reconstructed using a priori measurements of an incident ultrasound field with the spatially variant property. In [19], a 3D ultrasound imaging setup is shown that uses only one sensor. The sensor is equipped with an acoustic lens that yields individual objects in the ROI to be uniquely identifiable by the echo signals. In [20,21], specially tailored optoacoustic lenses are used to generate arbitrary acoustic fields and holograms. It was demonstrated that imaging with random ultrasound fields has the potential to improve the spatial resolution of ultrasound scanners beyond present limits.

In [22], we proposed a novel interference-based ultrasound imaging method, where instead of a traditional focused ultrasound beam, we use the transmission of an unfocused ultrasound wavefront of random interference. Here, we further improve the spatial and contrast resolutions of the interference-based imaging method by introducing a joint image reconstruction scheme. The new joint reconstruction scheme allows us to directly reconstruct high-resolution ultrasound images by utilizing radio frequency (RF) signals from all elements of the sensor array in a joint optimization problem. The joint reconstruction scheme provides superior contrast and image resolutions. According to the simulation study, the proposed joint reconstruction method leads to significant resolution improvements compared to different beamforming-based methods. The proposed method achieves a mean-squared error (MSE) of 0.00469, a peak signal-to-noise ratio (PSNR) of 33.3 dB, a signal-to-noise ratio (SNR) of 27.6 dB, and structural similarity (SSIM) of 0.998. This constitutes to a two-time resolution improvement over the previously proposed interference-based compound method. We share our simulation codes as an open-source repository. The link to the code repository can be found in Supplementary Materials.

The remainder of this paper is organized as follows. In Section 2, we describe the basic concept of ultrasound imaging using random interference and compressive sensing theory. In Section 3, we explain the proposed joint image reconstruction scheme. In Section 4, we provide the simulation and experimental results obtained. In Section 5, we discuss areas of future study. Finally, in Section 6 we summarize the contributions of this research.

## 2. Background

Traditionally, ultrasound interference has been treated as an undesired effect that degrades the quality of ultrasound images. We have recently shown that an ultrasound wavefront of random interference can be used as a means to differentiate between individual point scatterers in the ROI [23]. In this section, we review the effects of random interference and compressive sensing theory.

### 2.1. Effect of Random Interference

In conventional ultrasound imaging, beamforming is used to spatially divide the ROI into several scanlines. An ultrasound image is then acquired one scanline at a time by using beamforming to focus the transmit and receive ultrasound pulses. The image is reconstructed on the basis of the assumption that the received ultrasound signals consist of echoes reflected only from inhomogeneity within the given scanline. However, in practice, owing to the diffraction effect, the incident wavefront reflects from the medium within the scanline and its adjunct areas [23]. Owing to the imperfect coherence of echo signals, the beamformed signal is corrupted by interference patterns known as speckle noise [6,7,8]. Thus, in conventional focused methods, ultrasound interference is a highly undesired effect that degrades the image resolution.

Unlike conventional beamforming-based methods, we intentionally create an unfocused ultrasound wavefront of random interference, which yields spatial impulse responses of individual point scatterers in the ROI to be mutually incoherent [22]. Thus spatial resolution can be achieved by identifying individual point scatterers on the basis of their spatial impulse responses.

**Figure 1:**
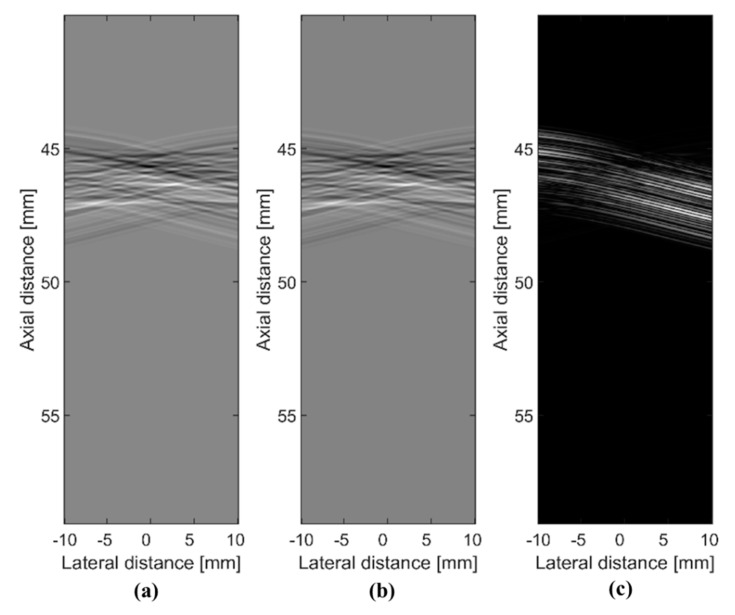
A wavefront of random interference is reflected from a group of scatterers and received echo signals visualized as grayscale images: (**a**) the group of point scatterers consists of four points located at lateral distances of −5, −1, 1, and 5 mm, respectively; (**b**) the point scatterers are located at lateral distances of −4.75, −1, 1, and 5 mm, respectively; (**c**) the absolute difference between image (**a**) and image (**b**).

Here, we demonstrate the effect of random interference, where a small change in the ROI incurs a significant difference in the received RF signals. In Figure 1, we show simulated RF signals obtained when a wavefront of random interference reflects from a group of scatterers [24,25,26]. In Figure 1, we show the echo signals of the incident wavefront of random interference reflected from the scatterers as grayscale images. In Figure 1a, the ROI includes a group of scatterers consisting of four points located at an axial distance of 45 mm and lateral distances of −5, −1, 1, and 5 mm, respectively. In Figure 1b, a group of point scatterers consists of four points located at lateral distances of −4.75, −1, 1, and 5 mm. In Figure 1b, the position of the first scatterer was changed from −5 mm to −4.75 mm. Figure 1c depicts the absolute difference between the images shown in Figure 1a,b. From Figure 1c, we can see that the effect of random interference yields a significant difference in the received echo signals even when we make a small change to the scatterer map. Using random interference and its effect on the scatterers in the ROI, we can design an imaging scheme where an ultrasound image is reconstructed using a priori information about wave propagation [18,22]. We represent the entire ROI as a set of individual spatial points. Then, the RF signals received at the sensor array can be decomposed into a set of echo signals reflected from individual point scatterers in the ROI. In a simulation study, a priori information can be acquired by generating RF signals for every point scatterer in the ROI. In the experimental study, a priori information can be generated from measurements of a plastic plane submerged into a water tank [22]. We summarize the steps used to acquire a priori information in Algorithm A1.

### 2.2. Compressive Sensing

With the proposed imaging method, we chose to use the compressive sensing (CS) theory because it provides a robust solution to underdetermined systems of linear equations. Originally, compressive sensing theory was introduced to reconstruct a signal using fewer measurements than that proposed by the Shannon–Nyquist sampling theorem [27,28,29]. However, later studies have shown that CS theory can be used to significantly improve the resolution of imaging modalities. During the past few decades, CS theory has been successfully used to improve the resolution of spectrometers, microscopes, and many other imaging modalities [30,31,32,33,34,35,36].

The concept of classical CS theory can be explained using a system of linear equations y=Ax=ΦΨx, where $x\in {\mathbb{R}}^{N}$ is a signal to be reconstructed, $A\in {\mathbb{R}}^{M\times N}$ is a specially constructed matrix consisting of a sensing matrix Φ and a sparsifying basis Ψ, and $y\in {\mathbb{R}}^{M}$ is a vector representing the signal acquired using a measuring instrument. The goal of CS is to reconstruct x from y and G when M<<N. In such a case, y=Ax is an underdetermined system because we have more unknowns than equations. The matrix A must characterize the transmission properties of the imaging system, therefore, it is called the transmission matrix. In the case of interference-based imaging, the transmission matrix characterizes the propagation properties of the wavefront of random interference. The CS theory guarantees the successful reconstruction of x under the following two conditions. The first condition requires the signal x to be sparse in some domain. The signal is called sparse if K non-zero values of the signal satisfy K<M<<N. The second condition requires a sensing matrix to be incoherent. The matrix is called incoherent when the cross-correlation of its columns is small. Many good algorithms were recently introduced to solve the *l*_1_-norm minimization problem and reliably reconstruct the signal of interest. In particular, we use the YALL1 MATLAB package solver [37].

## 3. Method

In [22], we proposed a new ultrasound imaging method based on random interference. In this paper, we further improve our results by proposing a joint image reconstruction scheme that combines RF signals from all elements of the sensor array in a single l_1_-norm minimization problem. As a result, the new proposed imaging scheme is capable of reconstructing ultrasound images with higher resolution.

### 3.1. Observation Model

**Figure 2:**
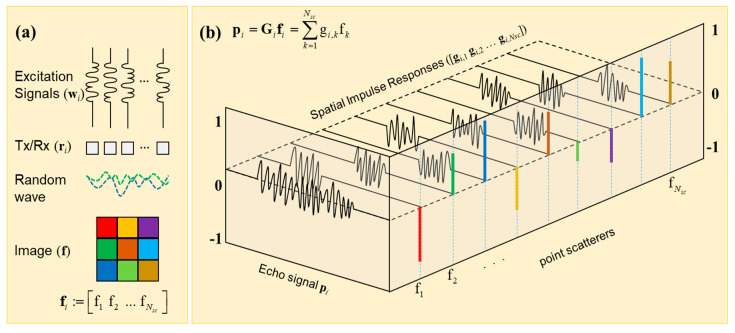
(**a**) the proposed imaging method utilizing a wavefront of random interference; (**b**) a diagram depicting the received echo signal as a sum of individual impulse responses of point scatterers.

Let us consider a linear transducer array with 128 piezoelectric elements. We let the vector $r_{i}$ for each $i\in \{1,2,\ldots ,N_{Rx}\}$ indicate the position of the array elements. In the proposed method, an acoustic wave is generated by simultaneously exciting all array elements using unique random signals. We use random excitation signals to drive the elements of the sensor array, which yields an ultrasound wavefront of random interference. We summarize the steps used to generate random excitation signals in Algorithm A1(Step 1). Let us consider a square image from 35 mm to 55 mm in the axial direction and from −10 mm to 10 mm in the lateral direction. Let us represent a medium within the ROI as a collection of point scatterers that are evenly distributed at equal distances d=0.25 mm apart. Thus, the image of interest consists of a total of $N_{Sc}=6561$ point scatterers. We let the vector $r_{k}$ for each $k\in \{1,2,\ldots ,N_{Sc}\}$ indicate the position of the *k*th scatterer in the ROI. We denote an ultrasound image as a vector $f_{i}:=\left[f_{1}{\;f}_{2}\;\ldots {\;f}_{N_{sc}}\right]$ for $i\in \{1,2,\ldots ,N_{Rx}\}$ and $k\in \{1,2,\ldots ,N_{Sc}\}$, where elements $f_{k}$ represent the inhomogeneity of the medium, which gives rise to the echo signal reflected off the scatterer at the *k*th spatial location. In Figure 2, we show a diagram of the proposed imaging method utilizing random interference. In Figure 2a, the random excitation signals are used to drive the transducer elements that yield an ultrasound wavefront of random interference. When the wavefront of random interference travels through the medium, its energy is reflected from the scatterers. Here, we consider a simple 3 × 3 image that can be represented as a vector $f_{i}:=\left[f_{1}{\;f}_{2}\;\ldots {\;f}_{N_{sc}}\right]$. In Figure 2b, we show that an echo signal $p_{i}$ received at the *i*th receiving element can be represented as the sum of individual impulse responses of the scatterer.

We can express an ultrasound echo signal received at the *i*th transducer element as

(1) $$p_{i}=G_{i}f_{i},$$

where $p_{i}\in {\mathbb{R}}^{M}$ is the received RF signal, $G_{i}\in {\mathbb{R}}^{M\times N}$ is the transmission matrix, and $f_{i}\in {\mathbb{R}}^{N}$ is a vectorized image, as observed by the *i*th array element. According to the principle of superposition [9], the received ultrasound echo signal $p_{i}$ is the sum of individual signals reflected off the scatterers $f_{i}$. Our goal is to find an estimate of the image f given the RF signals $p_{i}$ and transmission matrices $G_{i}$ for $i\in \{1,2,\ldots ,N_{Rx}\}$, this can be accomplished using CS theory [27,28,29].

### 3.2. Joint Image Reconstruction

In [22], we proposed an interference-based imaging method that used the compounding of several independently reconstructed images ${\hat{f}}_{i}$ given a set of echo signals $p_{i}$ and transmission matrices $G_{i}$. In this study, we propose a joint image reconstruction scheme that further improves the resolution of interference-based ultrasound imaging by directly reconstructing a high-resolution image.

When an ultrasound wavefront of random interference propagates through the medium, its energy is partially reflected and received at the transducer array. The RF signals carry information about the same object image $f_{\mathrm{Object}}$ observed at slightly different angles. In the interference-based joint image reconstruction method, we propose directly reconstructing a high-resolution image by utilizing all signals from the array in a single optimization problem.

First, to estimate the image f, we need to obtain transmission matrices that carry information about the propagation of the proposed ultrasound wavefront of random interference. We use the spatial impulse responses of individual point scatterers as a priori information. We can then reconstruct the ultrasound image using the following optimization problem:

(2) $${\hat{f}}_{i}:=\underset{f}{\arg\min}{\left\|f_{i}\right\|}_{1}\;\mathrm{subject}\;\mathrm{to}\;{\left\|G_{i}f_{i}-p_{i}\right\|}_{2}^{2}\leq \varepsilon .$$

In Equation (1), the image $f_{i}$ is reconstructed from an echo signal $p_{i}$ acquired at a single receiving channel *i.* In a single pulse-echo transmission, we use all elements of the array to receive the reflected echo signals. Therefore, we have 128 different versions of Equation (1), one for each receiving element in the array, as follows:

(3) $$\begin{array}{c} p_{1}=G_{1}f_{1}\\ p_{2}=G_{2}f_{2}\\ \vdots \\ p_{128}=G_{128}f_{128}. \end{array}$$

Owing to the effect of random interference, the received ultrasound signals $p_{i}$ and the transmission matrices $G_{i}$ for each $i\in \{1,2,\ldots ,N_{Rx}\}$ carry unique information about the image of interest $f_{i}$. In [22], an ultrasound image is reconstructed by applying the optimization problem in Equation (2) to the individual equations in Equation (3). The obtained images ${\hat{f}}_{i}$ for $i\in \{1,2,\ldots ,N_{Rx}\}$ are combined to form a high-resolution image as follows:

(4) $${\hat{f}}_{compound}=\left(\sum_{i=1}^{N_{Rx}}{\hat{f}}_{i}\right)/N_{Rx}.$$

However, a better and more accurate approach is to utilize the fact that the image of interest $f_{\mathrm{Object}}$ does not change depending on the receiving element number *i*. Therefore, the RF-signals $p_{i}$ for $i\in \{1,2,\ldots ,N_{Rx}\}$ include information about the same object image $f_{\mathrm{Object}}$. In such a case, we can use a joint image reconstruction scheme to further enhance the reconstruction accuracy and image resolution. As a simple solution to the joint reconstruction problem, we will next discuss a matrix inversion approach. First, we rearrange the measurement signals $p_{i}$ and transmission matrices $G_{i}$ in Equation (3) as follows:

(5) $$\left[\begin{array}{c} p_{1}\\ p_{2}\\ \vdots \\ p_{128} \end{array}\right]=\left[\begin{array}{c} G_{1}\\ G_{2}\\ \vdots \\ G_{128} \end{array}\right]\left[f_{\mathrm{Object}}\right].$$

For simplicity, let us use a subscript T to denote the tall matrix and tall vector in Equation (5) as follows:

(6) $$p_{T}=G_{T}f_{\mathrm{Object}}.$$

Then, a matrix inversion solution to Equation (6) can be written as

(7) $${G^{\prime }}_{T}p_{T}={G^{\prime }}_{T}G_{T}f_{\mathrm{Object}},$$

where $(\cdot {)}^{\prime }$ denotes a transpose. Then, the object image is equal to

(8) $$f_{\mathrm{Object}}={\left({G^{\prime }}_{T}G_{T}\right)}^{-1}{G^{\prime }}_{T}p_{T}.$$

In such a case, we can directly reconstruct a high-resolution image by utilizing information across the elements of the array. Similarly, we can use the optimization problem in Equation (2) to recover the image ${\hat{f}}_{\mathrm{Object}}$ as follows:

(9) $${\hat{f}}_{\mathrm{Object}}:=\underset{f}{\arg\min}{\left\|f_{\mathrm{Object}}\right\|}_{1}\;\mathrm{subject}\;\mathrm{to}\;{\left\|G_{T}f_{\mathrm{Object}}-p_{T}\right\|}_{2}^{2}\leq \varepsilon ,$$

where ${\left\|\cdot \right\|}_{1}$ denotes the *l*_1_-norm and ε≥0. To successfully reconstruct an object image ${\hat{f}}_{\mathrm{Object}}$, the transmission matrix in Equation (9) needs to be incoherent. We achieved incoherency by transmitting an ultrasound wavefront of random interference. We measure the incoherence of the transmission matrix G using the Gram matrix D=G*G. We assume that each column of the matrix G is normalized to one. The absolute value of the off-diagonal elements $\left|d_{i,j}\right|$ of matrix **D** represents a cross-correlation value between the corresponding pair of columns *i* and *j* of the matrix G.

## 4. Results

### 4.1. Simulation Study

In our simulation study, we use the Field II ultrasound software [24,25,26]. First, we simulate a linear transducer with 128 identical elements. Second, each array element was excited with a random sequence, as described in Section 2.1 and [22]. The central frequency was set to 3 MHz. The sampling frequency was set to 40 MHz.

**Figure 3:**
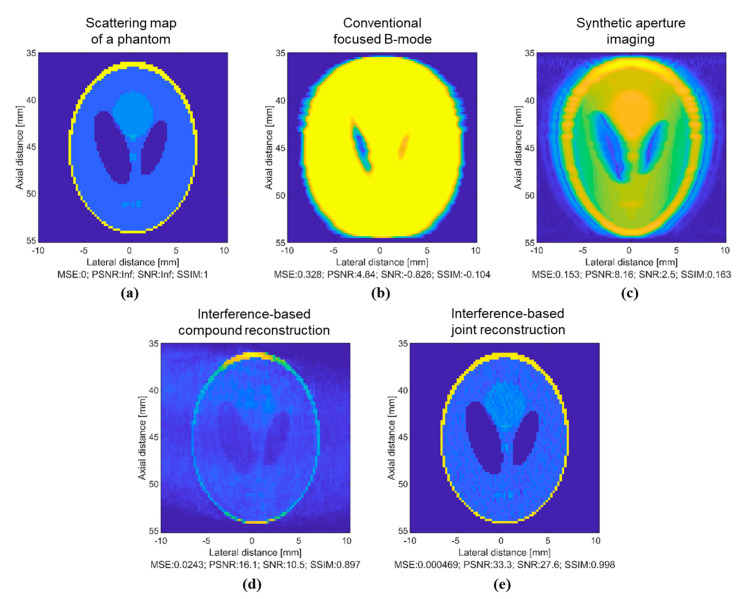
Simulation results: (**a**) a scattering map of the Shepp–Logan phantom; (**b**) an image reconstructed using a conventional focused B-mode; (**c**) an image reconstructed using synthetic aperture beamforming; (**d**) an image reconstructed using previously proposed interference-based compound reconstruction method; and (**e**) an image reconstructed using the proposed interference-based joint image reconstruction method.

To demonstrate the resolution capabilities of the newly proposed joint reconstruction scheme, we simulate a Shepp–Logan phantom. In Figure 3, we provide a side-by-side comparison of different reconstruction methods. The scattering map of the phantom is shown in Figure 3a. In Figure 3b, we show an image reconstructed using a conventional focused B-mode method. The image was reconstructed using 128 scanlines. The focal point was set to 45 mm depth. The image of the phantom appears with large sidelobes and the details of the phantom cannot be properly reconstructed. In Figure 3c, we show an image reconstructed using synthetic aperture imaging provided in the “Beamformation toolbox” [38]. The image was simulated using 65 emissions and beamformed with 180 lines. In Figure 3d, we show an image reconstructed using the interference-based compound method proposed in [23]. The reconstructed image features a more accurate location and intensity of the scatterers that precisely matches the original scatterer map of the phantom image. However, in the case of the compound method, the image in Figure 3d is corrupted by noise from the compounding operation. The presence of noise affects the visibility of small elements of the phantom at a lateral distance of 0 mm. In Figure 3e, we show an image reconstructed using the proposed joint image reconstruction scheme. The proposed joint reconstruction scheme allows us to successfully reconstruct a high-resolution image of the phantom. Figure 3e shows an image obtained using the proposed joint image reconstruction scheme. The image in Figure 3e has accurate spatial and contrast resolutions. The noise specific to the image in Figure 3d was eliminated, and the small details of the phantom at a lateral distance of 0 mm are accurately reconstructed. The image reconstructed using the newly proposed method exhibits less speckle noise and a more accurate representation of small components of the Shepp–Logan phantom. We compare the results from the Shepp–Logan phantom study in Table 1. The proposed interference-based joint reconstruction method, among all methods, has a lower MSE, higher PSNR and SNR, and an extremely high value for SSIM.

### 4.2. Experimental Study

In addition to the simulation results, we conducted an experimental study using a custom research ultrasound scanner supplied by Alpinion medical systems, Seoul, South Korea and a tissue-mimicking phantom Model 040GSE manufactured by CIRS, Virginia, USA. The ultrasound research equipment was equipped with an arbitrary wave generator (AWG) and dedicated memory to store the array of excitation signals. The AWG can store an array of size 128 × 2048 of eight-bit data, where each row is dedicated to the corresponding element of the sensor array. We used a linear transducer with 128 identical piezoelectric sensors (L3-12 manufactured by Alpinion, Seoul, South Korea). The central frequency was set to 3 MHz. Likewise, the sampling frequency was set to 40 MHz. 

Throughout this paper, we use the following parameters for different imaging techniques mentioned in the experimental study. In the case of the conventional focused B-mode, the center frequency was set to 6 MHz, the number of scanlines was set to 128, and the focal point was set at 5 cm depth. In the case of the plane-wave imaging, the center frequency was set to 6 MHz and the number of plane wave emissions was set to 7. Likewise, in the case of synthetic aperture imaging, we used 128 transmissions, one for each element in the sensor array, and the image consisted of 250 lines.

#### 4.2.1. Subarray Imaging

**Figure 4:**
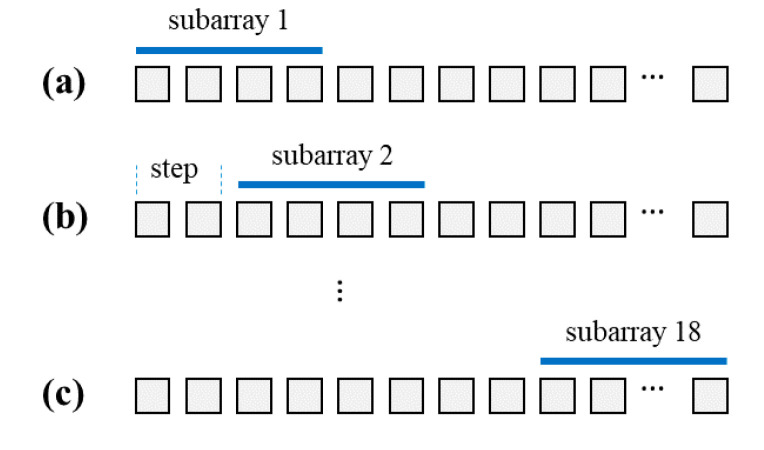
A diagram of selected subarrays.

In Equation (5), we suggest arranging echo signals $p_{i}$ and matrices $G_{i}$ into a tall vector $p_{T}$ and a tall matrix $G_{T}$, respectively. Although this is the best approach in practice, it is feasible only for small size problems. For example, in our experimental study, the dimension of RF signals is 128 × 3300, and the number of spatial points in ROI is 38,801. The dimension of the transmission matrix for a single *i*th receiving element is 3300 × 38,801. To use Equations (5) and (9), we would have to work with an extremely large tall matrix $G_{T}$. Here, we propose simplifying the joint image reconstruction method given in Section 3.2 and Equation (5) by reducing it to a set of smaller problems that divide the ROI into smaller imaging zones while still utilizing the joint reconstruction method. First, we perform 10 pulse-echo cycles each time with a different set of random excitation signals. In Figure 4, we show how RF signals from a single pulse-echo transmission can be rearranged into 18 different sets of subarrays signals. Then, we use subarray signals to reconstruct a subarray image using Equations (5) and (9).

**Figure 5:**
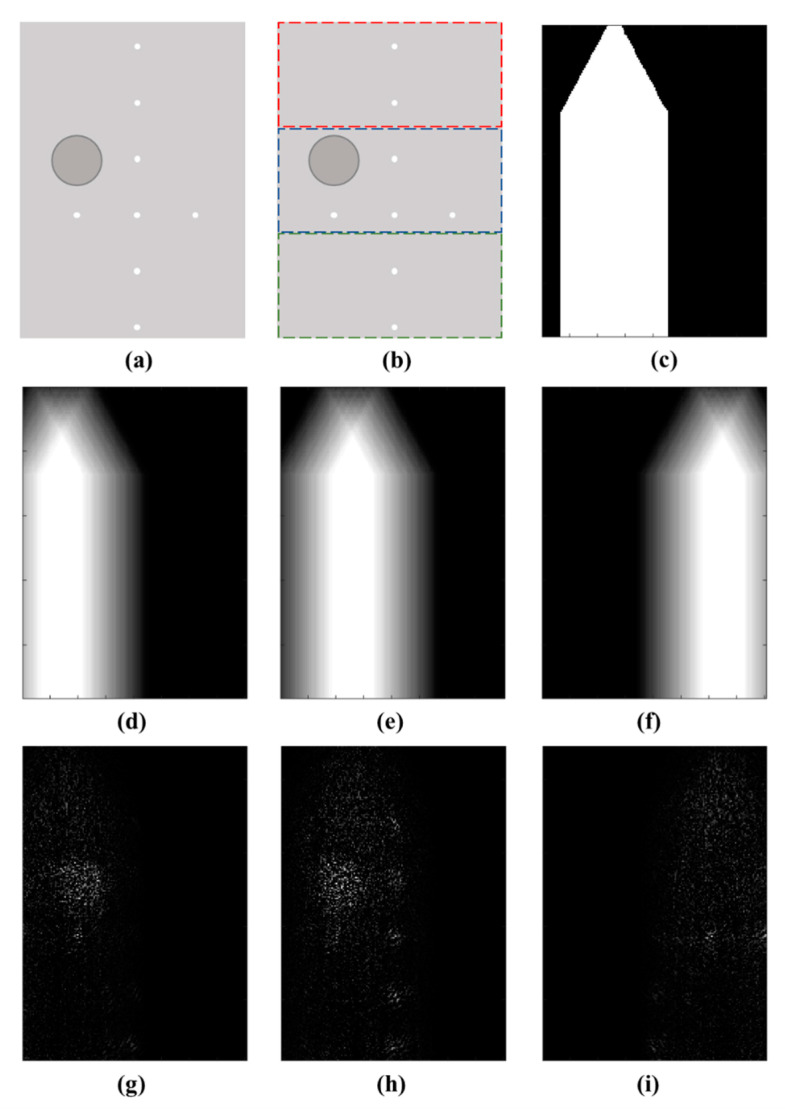
Experimental results: (**a**) an image of a tissue-mimicking phantom; (**b**) an image of a phantom with three dashed rectangles indicating the position of different imaging zones; (**c**) a diagram visualizing the angle of acceptance of a single receiving element in relation to the region of interest (ROI); (**d**–**f**) acceptance angle for subarrays; (**g**–**i**) low-resolution images obtained using joint reconstruction and subarrays.

In Figure 5a, we show a diagram of a tissue-mimicking phantom that represents the ROI. In Figure 5b, we show three rectangles that divide the ROI into three imaging zones. By reconstructing images separately for different imaging zones we can reduce the computational complexity. Likewise, in Figure 5c, we show the acceptance angle of a single piezo-electric sensor. Due to the sensor’s acceptance angle, not all points in the ROI contribute to the received RF signal. Therefore, echo signals reflected from spatial points that lay outside of the acceptance angle do not contribute to the RF signal acquired by the sensor. By removing columns that represent spatial responses of such points we can further reduce the computational complexity and increase image reconstruction accuracy. In Figure 5d–f, we show the acceptance angle for elements in the subarray. In Figure 5g–i, we show low-resolution images reconstructed for the selected subarrays using data acquired during a single pulse-echo transmission. We use data from 10 pulse-echo transmissions, each divided into 18 subarray configurations, and 3 imaging zones. In total, we have 540 subarray images that combined to form a final high-resolution image. 

#### 4.2.2. Single Wire Study

**Figure 6:**
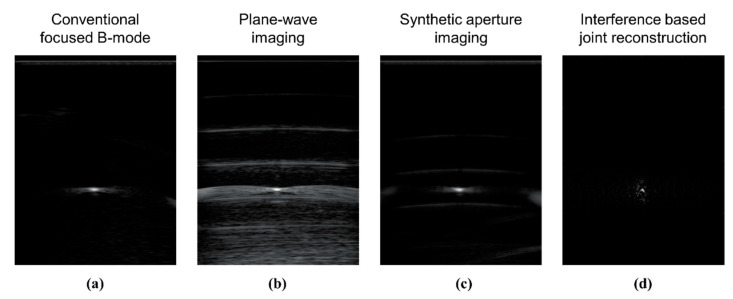
Experimental results using a nylon wire submerge into a water tank: (**a**) an image reconstructed using the conventional focused B-mode; (**b**) an image reconstructed using plane-wave imaging; (**c**) an image reconstructed using synthetic aperture imaging; (**d**) an image reconstructed using the interference-based joint reconstruction method using data from a single pulse-echo transmission.

In Figure 6, we compare images of a dental floss submerged into a water tank and reconstructed using different methods. In Figure 6a, we show an image reconstructed using a conventional focused B-mode method that can be found in all modern ultrasound systems. In Figure 6b, we show an image reconstructed using plane-wave imaging with 30 plane wave transmissions. In Figure 6c, we show an image reconstructed using synthetic aperture imaging. In Figure 6d, we show an image reconstructed using the proposed interference-based joint reconstruction method. The images in Figure 6a–c appear with side lobes caused by strong reflections of ultrasound waves that corrupt the image scanline during the beamforming operation. Meanwhile, the proposed method is capable of reconstructing a more accurate image of a wire without side lobes. The image in Figure 6d was reconstructed using data from a single pulse-echo transmission of a random interference wave.

#### 4.2.3. Tissue-Mimicking Phantom Study

**Figure 7:**
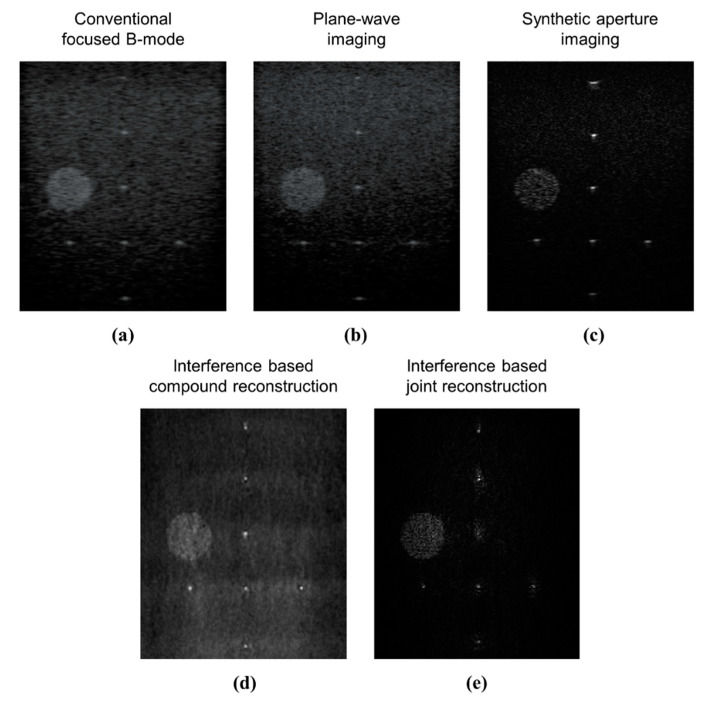
Comparison of different image reconstruction methods: (**a**) conventional focused B-mode; (**b**) plane-wave imaging; (**c**) synthetic aperture imaging; (**d**) interference-based compound reconstruction; and (**e**) proposed interference-based joint reconstruction.

In Figure 7, we compare images of a tissue-mimicking phantom reconstructed using different methods. In Figure 7a, we show images of a tissue-mimicking phantom reconstructed using a conventional focused B-mode method. In Figure 7b, we show an image reconstructed using plane-wave imaging. In Figure 7c, we show an image reconstructed using synthetic aperture imaging. In Figure 7d, we show an image reconstructed using the interference-based compound method. Likewise, in Figure 7e we show an image reconstructed using the proposed interference-based joint reconstruction method. From Figure 7, it can be observed that the proposed method reconstructs cyst and nylon wires with greater accuracy. The diameter of the nylon wires appears much closer to that of the original. Similarly, the speckle noise has been removed. The proposed joint image reconstruction method further improves the resolution of the interference-based ultrasound. The experimental results shown in Figure 7 are consistent with our simulation study shown in Figure 3. The image in Figure 7e was reconstructed using data from a ten pulse-echo transmission of random interference wave.

## 5. Discussion

Here, we share some thoughts on possible future ways to improve the resolution of interference-based imaging. The contribution of the proposed method is related to the area of image acquisition and reconstruction. Therefore, we did not use any signal/image post-processing techniques such as filtering or smoothing. However, in the future, it would be of great interest to see how modern speckle reduction techniques such as in [39,40] can be adapted to enhance the image quality of the proposed method. Likewise, in the proposed method, it is very important to obtain accurate a priori information. Therefore, it is of great interest to see if advanced beamforming algorithms such as those in [41,42,43] can be used to better estimate the spatial impulse responses.

The theoretical temporal resolution of the proposed method is equal to the time required to perform 10 pulse-echo transmissions. However, a significant amount of time is required to reconstruct high-resolution images. We use a PC equipped with Intel Xeon Gold 6240R, 256 GB RAM, and installed MATLAB environment. The maximum size of the array that can be efficiently processed using available PC is 40 × 3300 × 38,801. A single subarray image is reconstructed in about 110 s. The final high-resolution image is reconstructed in about 16 h. While it is a significant deviation from a common real-time ultrasound imaging, the proposed method achieves 250 μm resolution and has the potential to compete in some applications with 3 T magnetic resonance imaging MRI [44].

Using the proposed method, we were not able to obtain high-resolution in vivo images because biological tissue has a complex multi-layer structure that disperses the incident wavefront in various directions. Therefore, it is hard to generate transmission matrices that fit all possible cases of biological tissue. This is a topic of our ongoing research efforts. One can solve this issue with dynamically adjustable transmission matrices, where the ROI is divided into many thin layers. Then, the image reconstructed for the top layer can be used to estimate the transmission matrices for the next layers. Alternatively, a conventional B-mode image can be used as a reference to better estimate the transmission matrices.

## 6. Conclusions

In this work, we claim that by combining a novel interference-based image acquisition method with a joint image reconstruction algorithm we can achieve very accurate ultrasound images at 250 μm resolution with strong SNR and precise SSIM. The proposed joint reconstruction method uses echo signals across all array elements to directly estimate a high-resolution image. The proposed method does not require a focused ultrasound beam, thereby removing the speckle noise caused by diffraction. The interference-based method is different from conventional approaches that use the strength of the echo signals to visualize details of imaged objects. Instead, an intentionally created wavefront of random interference and a *l*_1_-norm minimization algorithm are used to visualize the object by identifying individual spatial impulse responses of the scatterers. Using simulation and experimental results, we have shown that the joint image reconstruction method improves the resolution of ultrasound images.

## Figures and Tables

**Table 1:** Mean-squared error (MSE), peak signal-to-noise ratio (PSNR), signal-to-noise ratio (SNR), and structural similarity index (SSIM) values for conventional and proposed imaging methods.

Method	MSE	PSNR	SNR	SSIM
1. Conventional focused B-mode	0.328	4.84	−0.826	−0.104
2. Synthetic aperture beamforming	0.153	8.16	2.5	0.163
3. Interference-based compound reconstruction [15]	0.0243	16.1	10.5	0.897
4. Interference-based joint reconstruction	0.000469	33.3	27.6	0.998

## Supplementary Materials

Simulation codes and instructions to reproduce the results of this study are available at https://github.com/infonetGIST/Sensors-Ultrasound-Random-Interference.

## Appendix A

**Algorithm A1.** Pseudocode for random interference and joint image reconstruction.
**Step 1: Random excitation signals:** **Input:***Number-of-Elements, Binary-Sequence-Length, Half-Sine-Chirp* **Output:***Random-Excitation-Signals* **Initialization:***Number-of-Elements := 128; Binary-Sequence-Length := 13; Half-Sine-Chirp;* **Begin Algorithm** *Random-Excitation-Signals* := [] **for** (*Array-Element := 1* **to** *Number-of-Elements*) **do** *Binary-Sequence* := Generate random sequence of [1 −1] of length 13 *Peaks-and-Valleys* := [] **for** (Sequence-Element := 1 **to** *Binary-Sequence-Length*) **do** *Peak-or-Valley* := *Half-Sine-Chirp * Binary-Sequence*[*Sequence-Element*] *Peaks-and-Valleys* := [*Peaks-and-Valleys*, *Peak-or-Valley*] **end for** *Random-Excitation-Signals*[*Array-Element*, :] := *Peaks-and-Valleys* **end for** **return** *Random-Excitation-Signals* **Step 2: Region of Interest (ROI)** **Input:** *ROI-Lateral, ROI-Axial, Number-of-Elements, Element-Width, Element-Height, Kerf* **Output:** *Spatial-Points* **Initialization:** *ROI-Lateral* := [−10, 10]; *ROI-Axial* := [35, 55]; *Number-of-Elements* := 128; *Element-Height* := 4.5 mm; *Element-Width* := 0.27 mm; *Kerf* := 0.03 mm; *Resolution* := 0.25 mm; **Begin Algorithm** *Spatial-Points* := [] *Axial-Points* := [*ROI-Lateral* [1] : *Resolution* : *ROI-Lateral*[2]] *Lateral-Points* := [*ROI-Axial*[1] : *Resolution* : *ROI-Axial*[2]] **for** (*point-ax* := 1 **to** length(*Axial-Points*)) **do** **for** (*point-la* := 1 **to** length(*Lateral-Points*)) **do** *Spatial-Points* := := [*Spatial-Points* ([*Axial-Points*(*point-ax*), 0, *Lateral-Points*(*point-la*)])] **end for** **end for** **return** *Spatial-Points* **Step 3: A priori information (Transmission matrices)** **Input:** *Number-of-Elements, Random-Excitation-Signals* **Output:** *Transmission-Matrix* **Initialization:** *Number-of-Elements* := 128; *Random-Excitation-Signals*; *Spatial-Points*; //The following code requires Field ii ultrasound simulation software (calc_scat_multi function). **Begin Algorithm** Define simulation environment Define transducer array *Tx* Assign *Random-Excitation-Signals* to the elements of sensor array. **for** (*Array-Element* := 1 **to** *Number-of-Elements*) **do** *Transmission-Matrix* := [] **for** (*Spatial-Point* := 1 **to** length(*Spatial-Points*)) **do** //Simulate RF-signals reflected off a single scatterer at Spatial-Point //The RF-signals can be generated in advance [*v*, *t*] := calc_scat_multi(*Tx*, *Tx*, *Spatial-Point*, 1) *Transmission-Matrix* := [*Transmission-Matrix*, *v*[: , *Array-Element*]] **end for** **return** *Transmission-Matrix*//Transmission matrix for an *Array-Element* **end for** **Step 4: Joint image reconstruction** **Input:** *Transmission-Matrix*, *Measurements* **Output:** *Hires-Image* **Initialization:** *Transmission-Matrices*; *Measurements*; *Spatial-Points*; //The following code requires Yall1 algorithm (yall1 function) **Begin Algorithm** *Tall-Matrix* := [] *Tall-Measurements* := [] **for** (*Array-Element* := 1 **to** *Number-of-Elements*) **do** *Tall-Matrix* := [*Tall-Matrix*, *Transmission-Matrix*[*Array-Element*]] *Tall-Measurements* := [*Tall-Measurements*, *Measurements*[*Array-Element*]] **end for** *Hires-Image* := yall1(*Tall-Matrix*, *Tall-Measurements*)

## References

[1] Steinberg B. (1992). Digital beamforming in ultrasound. IEEE Trans. Ultrason. Ferroelectr. Freq. Control..

[2] Johnson J., Karaman M., Khuri-Yakub P. (2002). Synthetic phased array image formation and restoration. IEEE Int. Conf. Acoust. Speech Signal Process..

[3] Szabo T. (2014). Diagnostic Ultrasound Imaging: Inside Out.

[4] Ng A., Swanevelder J. (2011). Resolution in ultrasound imaging. Contin. Educ. Anaesth. Crit. Care Pain.

[5] Maznev A.A., Wright O.B. (2017). Upholding the diffraction limit in the focusing of light and sound. Wave Motion.

[6] Goodman J.W. (1976). Some fundamental properties of speckle. J. Opt. Soc. Am..

[7] Burckhardt C.B. (1978). Speckle in ultrasound B-mode scans. IEEE Trans. Sonics Ultrason..

[8] Abbott J.G., Thurstone F.L. (1979). Acoustic speckle: Theory and experimental analysis. Ultrasound Imag..

[9] Duarte-Salazar C.A., Castro-Ospina A.E., Becerra M.A., Delgado-Trejos E. (2020). Speckle Noise Reduction in Ultrasound Images for Improving the Metrological Evaluation of Biomedical Applications: An Overview. IEEE Access.

[10] Ortiz S.H.C., Chiu T., Fox M.D. (2012). Ultrasound image enhancement: A review. Biomed. Signal Process. Control..

[11] Dutt V., Greenleaf J. (1996). Adaptive speckle reduction filter for log-compressed B-scan images. IEEE Trans. Med Imaging.

[12] Ranjani J.J., Chithra M.S. (2015). Bayesian denoising of ultrasound images using heavy-tailed Levy distribution. Image Process. IET.

[13] Viessmann O.M., Eckersley R.J., Christensen-Jeffries K., Tang M.X., Dunsby C. (2013). Acoustic super-resolution with ultrasound and microbubbles. Phys. Med. Biol..

[14] Yu J., Lavery L., Kim K. (2018). Super-resolution ultrasound imaging method for microvasculature in vivo with a high temporal accuracy. Sci. Rep..

[15] Song P., Trzasko J.D., Manduca A., Huang R., Kadirvel R., Kallmes D.F., Chen S. (2018). Improved Super-Resolution Ultrasound Microvessel Imaging With Spatiotemporal Nonlocal Means Filtering and Bipartite Graph-Based Microbubble Tracking. IEEE Trans. Ultrason. Ferroelectr. Freq. Control..

[16] Nowicki A., Klimonda Z., Lewandowski M., Litniewski J., Lewin P., Trots I. (2006). Comparison of sound fields generated by different coded excitations—Experimental results. Ultrasonics.

[17] Song J.H., Kim S., Sohn H.-Y., Song T.-K., Yoo Y.M. (2010). Coded excitation for ultrasound tissue harmonic imaging. Ultrasonics.

[18] Clement G.T., Huttunen J., Hynynen K. (2005). Superresolution ultrasound imaging using back-projected reconstruction. J. Acoust. Soc. Am..

[19] Kruizinga P., Van Der Meulen P., Fedjajevs A., Mastik F., Springeling G., De Jong N., Bosch J.G., Leus G. (2017). Compressive 3D ultrasound imaging using a single sensor. Sci. Adv..

[20] Brown M.D., Nikitichev D.I., Treeby B.E., Cox B.T. (2017). Generating arbitrary ultrasound fields with tailored optoacoustic surface profiles. Appl. Phys. Lett..

[21] Brown M.D., Cox B., Treeby B.E. (2020). Stackable acoustic holograms. Appl. Phys. Lett..

[22] Ni P., Lee H.-N. (2020). High-Resolution Ultrasound Imaging Using Random Interference. IEEE Trans. Ultrason. Ferroelectr. Freq. Control..

[23] Cox B., Beard P. (2015). Imaging techniques: Super-resolution ultrasound. Nature.

[24] Jensen J.A. (1999). Linear description of ultrasound imaging systems. Notes for the International Summer School on Advanced Ultrasound Imaging.

[25] Jensen J.A., Svendsen N.B. (1992). Calculation of pressure fields from arbitrarily shaped, apodized, and excited ultrasound transducers. IEEE Trans. Ultrason. Ferroelectr. Freq. Control..

[26] Jensen J.A. (1996). Field: A program for simulating ultrasound systems. Med Biol. Eng. Comput..

[27] Candes E., Romberg J., Tao T. (2006). Robust uncertainty principles: Exact signal reconstruction from highly incomplete frequency information. IEEE Trans. Inf. Theory.

[28] Candès E., Romberg J. (2006). Quantitative robust uncertainty principles and optimally sparse decompositions. Found. Comput. Math..

[29] Donoho D. (2006). Compressed sensing. IEEE Trans. Inf. Theory.

[30] Oliver J., Lee W., Park S., Lee H.-N. (2012). Improving resolution of miniature spectrometers by exploiting sparse nature of signals. Opt. Express.

[31] Jang H., Yoon C., Chung E., Choi W., Lee H.-N. (2014). Speckle suppression via sparse representation for wide-field imaging through turbid media. Opt. Express.

[32] Lee W.-B., Jang H., Park S., Song Y.M. (2016). COMPU-EYE: A high resolution computational compound eye. Opt. Express.

[33] Baboulaz L., Dragotti P.L. (2009). Exact Feature Extraction Using Finite Rate of Innovation Principles with an Application to Image Super-Resolution. IEEE Trans. Image Process..

[34] Mishra K.V., Cho M., Kruger A., Xu W. (2015). Spectral Super-Resolution with Prior Knowledge. IEEE Trans. Signal Process..

[35] Jung H., Sung K., Nayak K.S., Kim E.Y., Ye J.C. (2009). k-t FOCUSS: A general compressed sensing framework for high resolution dynamic MRI. Magn. Reson. Med..

[36] Herman M.A., Strohmer T. (2009). High-Resolution Radar via Compressed Sensing. IEEE Trans. Signal Process..

[37] Yang F., Zhang Y. (2011). Alternating direction algorithms for `l1-problems in compressive sensing. SIAM J. Sci. Comput..

[38] Nikolov S.I. (2000). User’s Guide to the Beamformation Toolbox.

[39] Rekha C.K., Manjunathachari K., Rao G.V.S. Speckle noise reduction in 3D ultrasound images—A review. Proceedings of the 2015 International Conference on Signal Processing and Communication Engineering Systems.

[40] Coupe P., Hellier P., Kervrann C., Barillot C. (2009). Nonlocal means-based speckle filtering for ultrasound images. IEEE Trans. Image Process..

[41] Avanaki K., Zafar M., Mozaffarzadeh M., Hariri A., Haung X., Orooji M., Avanaki K. (2018). A Novel Dictionary-Based Image Reconstruction for Photoacoustic Computed Tomography. Appl. Sci..

[42] Mozaffarzadeh M. (2018). Linear-array photoacoustic imaging using minimum variance-based delay multiply and sum adaptive beamforming algorithm. J. Biomed. Opt..

[43] Merabet L., Robert S., Prada C. (2019). 2-D and 3-D Reconstruction Algorithms in the Fourier Domain for Plane-Wave Imaging in Nondestructive Testing. IEEE Trans. Ultrason. Ferroelectr. Freq. Control..

[44] Van Reeth E., Tham I.W.K., Tan C.H., Poh C.L. (2012). Super-resolution in magnetic resonance imaging: A review. Concepts Magn. Reson. Part A.

